# Effects of maximum dose on local control after stereotactic body radiotherapy for oligometastatic tumors of colorectal cancer

**DOI:** 10.1371/journal.pone.0313438

**Published:** 2025-01-03

**Authors:** Su Jin Kang, Jongmoo Park, Gyu-Seog Choi, Jong Gwang Kim, Jun Seok Park, Hye Jin Kim, Jin Ho Baek, Byung Woog Kang, An Na Seo, Shin-Hyung Park, Bong Kyung Bae, Min Kyu Kang, Soo Yeun Park

**Affiliations:** 1 Colorectal Cancer Center, Kyungpook National University Chilgok Hospital, School of Medicine, Kyungpook National University, Daegu, Republic of Korea; 2 Department of Radiation Oncology, School of Medicine, Kyungpook National University, Daegu, Republic of Korea; 3 Department of Oncology/Hematology, School of Medicine, Kyungpook National University, Daegu, Republic of Korea; 4 Department of Pathology, School of Medicine, Kyungpook National University, Kyungpook National University Chilgok Hospital, Daegu, Republic of Korea; MD Anderson Center, UNITED STATES OF AMERICA

## Abstract

This study aimed to identify radiotherapy dosimetric parameters related to local failure (LF)-free survival (LFFS) in patients with lung and liver oligometastases from colorectal cancer treated with stereotactic body radiotherapy (SBRT). We analyzed 75 oligometastatic lesions in 55 patients treated with SBRT between January 2014 and December 2021. There was no constraint or intentional increase in maximum dose. LF was defined as the progression of the treated lesion until the last follow-up or death. The dose distributions were recalculated using Monte Carlo-based algorithms. The significance of the planning target volume (PTV) biologically effective dose (BED) 10s (D2, D95, D98, Dmean) in LFFS was evaluated using Cox regression, considering sex, age, primary cancer, tumor site, oligometastatic status, multiplicity, and either tumor size or one of the volume parameters. LF occurred in 23.4% of the lesions. Lesions showing LF received significantly lower PTV D2 (146 ± 21 vs. 164 ± 23, *p* = 0.006). Multivariate analysis revealed that PTV D2 (< 159 Gy_10_ vs. ≥ 159 Gy_10_) was the sole dosimetric parameter associated with LFFS. Tumors equal to or larger than the median size/volume yet receiving < 159 Gy_10_ of PTV D2 showed the lowest LFFS following stratification by median PTV D2 combined with tumor size or volume parameters. The maximum dose (PTV D2) was significantly associated with LFFS after SBRT for lung and liver oligometastases from colorectal cancer. Increasing the maximum dose may be beneficial for managing larger tumors.

## Introduction

Metastatic colorectal cancer presents a complicated clinical situation and often requires multidisciplinary management. Oligometastatic disease, defined as the presence of 1–5 metastatic lesions, is managed with direct local treatment to potentially achieve disease control or eradication [1, 2]. Although surgery has long been the standard treatment option, other local therapies can be alternatives in patients with lesions unsuitable for resection or comorbidities hindering surgery [1, 3–5].

Stereotactic body radiotherapy (SBRT) delivers high-dose radiation and demonstrates high local control rates of 80–90% for primary or metastatic tumors [6]. A radiation dose ensuring a local control probability of approximately 90% is suggested to be approximately 100 Gy_10_ of biologically effective dose with an α/β ratio of 10 (BED10) for primary lung tumors. However, local control rates after SBRT for metastatic colorectal cancer highly vary [7]. A meta-analysis revealed that oligometastatic tumors from colorectal cancer were more radioresistant compared with those from non-colorectal primary tumors, indicating the need for dose escalation [8]. In patients with metastatic colorectal cancer, a higher BED10 prescription dose achieves higher local control after SBRT in various retrospective studies: ≥ 100 Gy_10_ [9–12], ≥ 120–132 Gy_10_ [13–15], or ≥ 150 Gy_10_ [16, 17]. Although target doses significantly differ according to the combination of several factors [18–20], most of these studies have not provided sufficient dosimetric information on dose calculation methods or dosimetric parameters.

Therefore, we aimed to evaluate which radiotherapy dosimetric parameters were associated with local failure (LF)-free survival (LFFS) after SBRT for liver and lung oligometastases from colorectal cancer using dosimetric data calculated using Monte Carlo-based algorithms to guide for SBRT treatment planning.

## Materials and methods

### Patients

This retrospective study was conducted by a multidisciplinary team of specialists in radiation oncology, surgery, medical oncology, pathology, radiology, and nuclear medicine. With the growing use of SBRT in patients with osteoblastic metastatic disease, the team initiated research to establish an institutional protocol for optimal dose prescription. The study assessed the dose parameters and oncologic outcomes in previously treated patients. We reviewed the medical records of 116 patients treated with SBRT for liver or lung oligometastases from colorectal adenocarcinoma at a single tertiary university hospital between January 2014 and December 2021. The data was accessed for research purposes between 12 April 2024 and 21 April 2024 for processing and analyzing the raw data and conducting statistical analysis. Individual data was processed after collection; identifying information was encrypted. Therefore, the authors were unable to identify individual participants. The patients who met the following exclusion criteria were excluded: (1) all lesions not simultaneously or sequentially treated with local treatment modalities (n = 21), (2) non-oligometastatic disease at the time of each SBRT session (n = 8), (3) no imaging follow-up after SBRT (n = 5), (4) incomplete treatment of scheduled SBRT (n = 2), (5) inability to analyze dosimetric parameters for each lesion (n = 2), (6) BED10 of the prescription dose < 100 Gy_10_ (n = 2), and (7) surgical resection after SBRT without the evidence of progression (n = 1). Oligometastatic disease was defined as one to five metastatic lesions safely treated in patients with a controlled primary tumor [2]. Oligometastasis was defined as metastatic tumors present at the time of diagnosis or recurrence; oligo-progression was defined as metastatic lesions that progressed during or at the end of the systemic therapy. Finally, 75 oligometastatic lesions in 55 patients were evaluated in this study. This study was approved by the Institutional Review Board of our Hospital. The requirement for informed consent was waived due to the retrospective nature of this study.

### Radiotherapy

**Fig 1:**
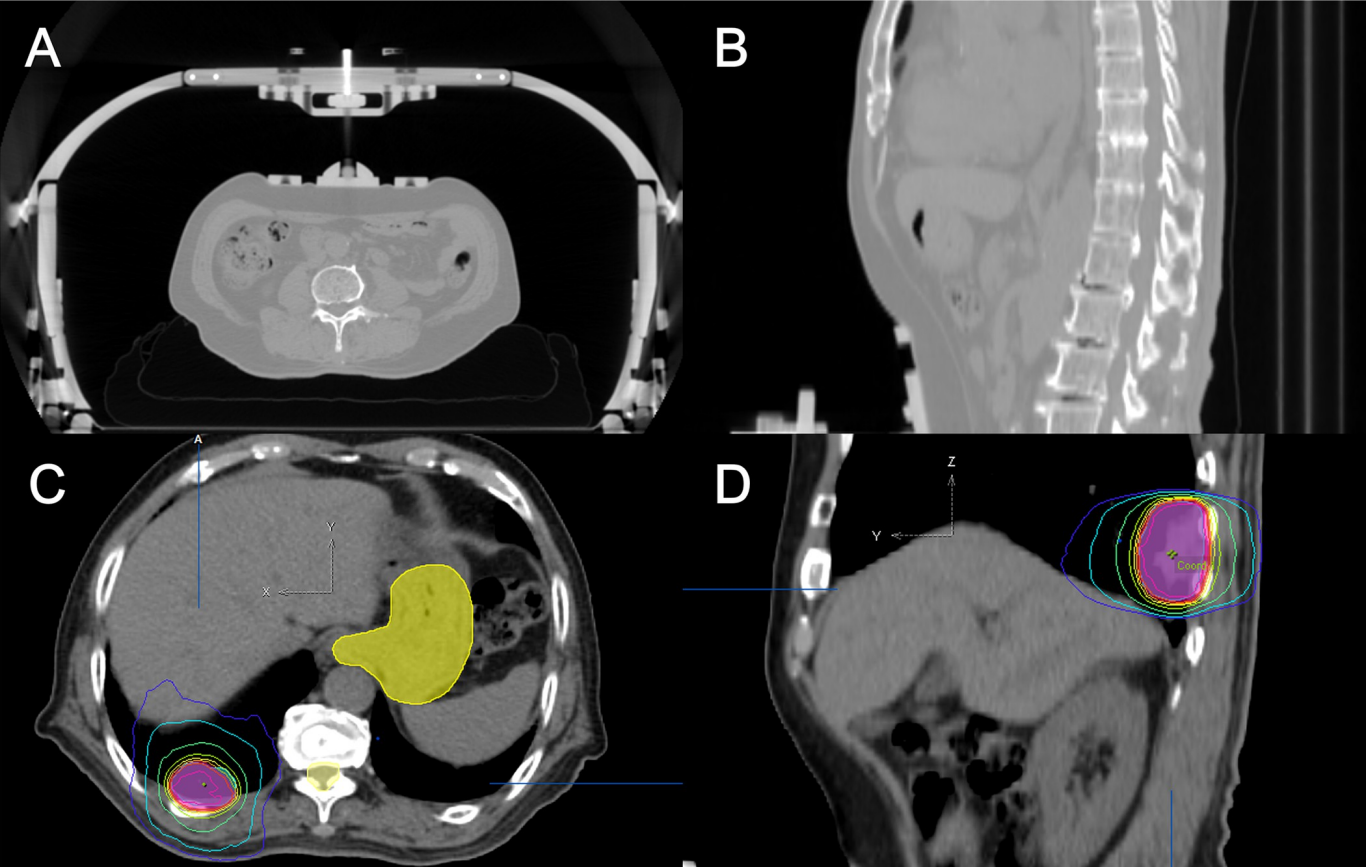
Lung SBRT planning using an abdominal compression device. The simulation images acquired with the abdominal compression technique (A, B). The treatment plan was calculated using iPlan software for the VERO system, as shown in (C, D), illustrating dose distribution and target coverage.

The patients were immobilized in the supine position. Computed tomography (CT) images were acquired using a respiratory gating or abdominal compression device (Fig 1). Gross tumor volume (GTV) was defined as the tumor observed in imaging studies. Internal tumor volume (ITV) was defined as the sum of the GTVs in each set of CT scans. Planning target volume (PTV) was usually generated with a 5-mm margin from the ITV. The radiation dose was usually prescribed to cover 95% of the PTV. There was no constraint or intentional increase in the maximum dose (Dmax), with modifications made based on the proximity of organs at risk and physician’s clinical judgment. S1 Table shows the dose-fractionation schedules according to tumor location. Respiratory management methods and beam delivery techniques are summarized in Table 1.

**Table 1:** Respiratory management methods and beam delivery techniques.

Beam delivery techniques	Respiratory management methods			
	Encompassing	Gating	Tracking	Abdominal compression
Dynamic conformal arc	58	1	0	5
Static conformal beam	0	0	4	0
Intensity-modulated beam	4	1	1	1

Before collecting the dosimetric parameters, dose distributions derived from the pencil beam algorithm or AAA were recalculated using XVMC or AXB to address the dose bias inherent in different dose calculation algorithms and better represent the actual tumor doses. Subsequently, the doses covering 2%, 95%, and 98% of the PTV (D2, D95, and D98) and the mean dose (Dmean) of the PTV were converted into BED10 values using the following equation:

$$BED10=nd(1+\frac{d}{\alpha /\beta })$$

where n was the total number of treatment fractions, d the dose delivered per fraction in Gy, and α/β the dose at which the contributions of the linear and quadratic cell-killing effects are equal in the linear-quadratic model. A α/β ratio of 10 Gy was assumed for the tumors in this study.

### Systemic treatment

Regarding colon cancer, doublet chemotherapy (FOLFOX or FOLFIRI) with or without targeted agents was used as the first- or second-line treatment, while targeted agents were incorporated in subsequent lines of therapy. The patients with initially diagnosed locally advanced rectal cancer received 5-fluorouracil or capecitabine concurrently with preoperative radiotherapy, followed by adjuvant capecitabine or FOLFOX. In patients with rectal cancer with initially M1 or recurrent disease, systemic regimens similar to those used for colon cancer were applied as subsequent lines of chemotherapy.

### Statistical analyses

Categorical variables are presented as frequencies (percent) and were compared using Pearson’s *x*^*2*^ test or Fisher’s exact test. Continuous variables are presented as means (± standard deviations) and medians (interquartile ranges), which were compared using Student’s t-test or the Mann–Whitney U test. The correlation between two continuous variables was analyzed using Pearson’s correlation test. LF was defined as progression of the treated lesion until the last follow-up or death, regardless of disease progression at other sites. The LFFS rate was calculated from the first day of SBRT to the event or last follow-up using the Kaplan–Meier method. Overall survival (OS) rate was calculated from the first day of SBRT to the date of death or the last follow-up, considering the first SBRT session in patients treated with multiple SBRT sessions. Risk factors for LFFS were determined using the log-rank test and the Cox regression hazards model with backward elimination after stratification by the median values of dosimetric parameters, tumor size, and target volumes. All statistical analyses were performed using R Statistics (version 4.3.2, The R Foundation for Statistical Computing, Vienna, Austria). A *p*-value of < 0.05 was considered statistically significant.

## Results

### Patient characteristics

The patient characteristics based on the first SBRT session are summarized in Table 2. The median age of the patients was 71 (range, 27–91) years. The male:female ratio was 35:20. The primary tumor locations were the colon and rectum in 41.8% and 58.2% of the patients, respectively. KRAS mutations were observed in 52.7% of the patients, whereas microsatellite instability accounted for 1.8%. The 2-year OS was 80.0%, with a median follow-up period of 31.7 (range, 6.5–76.8) months. There were no cases of mucinous adenocarcinoma or signet ring cell carcinoma.

**Table 2:** Patient characteristics.

	Number (N = 55)
Sex	
Male	35 (63.6%)​
Female	20 (36.4%)
ECOG performance status	
0–1	31 (56.4%)
2–3	19 (43.6%)
Primary tumor stie	
Colon	23 (41.8%)​
Rectum	32 (58.2%)​
Tumor differentiation	
Well or moderately	48 (87.3%)​
Poorly	7 (12.7%)​
KRAS mutation	
No	16 (29.1%)
Yes	29 (52.7%)
unknown	10 (18.2%)
Microsatellite instability	
No	36 (65.5%)
Yes	1 (1.8%)
unknown	18 (32.7%)

*Abbreviations*: ECOG, Eastern Cooperative Oncology Group

### Tumor characteristics

Tumor characteristics based on each lesion are summarized in Table 3; tumor locations were the lungs in 86.7% and liver in 13.3% of the patients. Among the patients, 34 (45.3%) received 1 line of systemic treatment, 21 (28.0%) received 2 lines, 6 (8.0%) received 3 lines, and 1 patient (1.3%) received 4 lines. Thirteen patients did not undergo systemic treatment. The median tumor size was 11 (range, 4–45) mm. The median volumes of GTV and PTV were 1.0 (range, 0.03–25.9) cc and 10.2 (range, 3.7–88.7) cc, respectively. The median doses of D2, D95, D98, and Dmean of the PTV were 159 Gy_10_ (range, 102–238), 113 Gy_10_ (range, 86–157), 106 Gy_10_ (range, 80–146), and 135 Gy_10_ (range, 98–194), respectively, with a significant correlation between them (S1 Fig).

**Table 3:** Tumor and treatment characteristics.

	N	(%)
Oligometastatic disease		
De novo	27	(36.0%)
Repeat	37	(49.3%)
Induced	11	(14.7%)
Tumor site		
Lung	65	(86.7%)
Liver	10	(13.3%)
Multiplicity		
Single	37	(49.3%)
Multiple	38	(50.7%)
Systemic treatment		
1st lines		34 (45.3%)
2nd lines		21 (28.0%)
3rd lines		6 (8.%)
4th lines		1 (1.3%)
No systemic treatment		13 (17.3%)
Tumor size (mm)	11	(4–45)
GTV volume (cc)	1.0	(0.3–25.9)
PTV volume (cc)	10.2	(3.7–88.7)
PTV D2 (Gy_10_)	159	(102–239)
PTV D95 (Gy_10_)	113	(86–157)
PTV D98 (Gy_10_)	106	(80–146)
PTV Dmean (Gy_10_)	135	(98–194)

*Abbreviations*: GTV, gross tumor volume; ITV, interval tumor volume; PTV, planning target volume

### Prognostic factors for local failure

**Fig 2:**
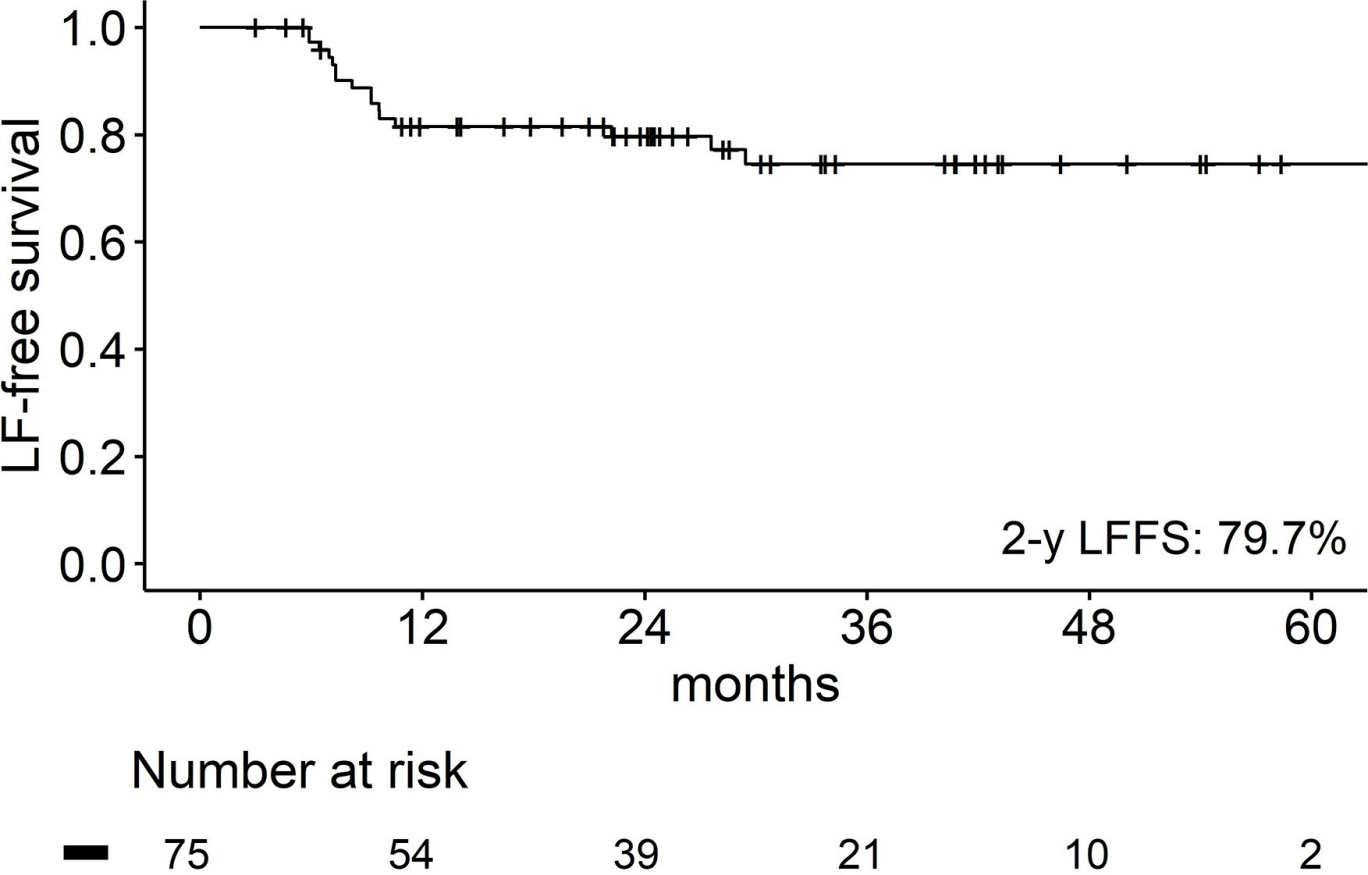
Kaplan–Meier curve of local failure-free survival.

LF developed in 16 (21.3%) out of 75 treated lesions with the median follow-up period of 24.3 (range, 3.0–74.5) months. The 2-year LFFS rate was 79.7% (Fig 2). The tumor characteristics based on LF are summarized in Table 4. Tumors showing LF had significantly larger tumor in terms of tumor size (*p* = 0.002), GTV volume (*p* = 0.001), ITV volume (*p* < 0.001), and PTV volume (*p* < 0.001) and received significantly lower PTV D2 (*p* = 0.006). PTV D95, D98, and Dmean did not differ between tumors with and without LF.

**Table 4:** Tumor characteristics based on the presence of local failure.

	Local failure		
	Absent (n = 59)	Present (n = 16)	*P*
Primary tumor			0.226
Colon	27 (45.8%)	4 (25.0%)	
Rectum	32 (54.2%)	12 (75.0%)	
Tumor site			0.206
Lung	53 (89.4%)	12 (75.0%)	
Liver	6 (10.6%)	4 (40%)	
Oligometastatic type			
Oligometastasis	44 (74.6%)	9 (56.2%)	0.215
Oligoprogression	15 (25.4%)	7 (43.8%)	
Multiplicity			1.000
Single	30 (50.8%)	8 (50.0%)	
Multiple	29 (49.2%)	8 (50.0%)	
Tumor size (mm)	10 (6–15)	17 (12–22)	0.002
GTV volume (cc)	0.6 (0.3–2.1)	5.2 (1.5–11.7)	0.001
ITV volume (cc)	1.0 (0.7–3.4)	7.5 (3.8–15.6)	<0.001
PTV volume (cc)	8.2 (6.1–13.5)	27.4 (15.6–44.2)	<0.001
PTV D2 (Gy_10_)	164 (141–187)	146 (125–167)	0.006
PTV D95 (Gy_10_)	113 (105–120)	112 (106–117)	0.628
PTV D98 (Gy_10_)	108 (99–114)	105 (101–112)	0.742
PTV Dmean (Gy_10_)	137 (129–144)	132 (121–139)	0.142

*Abbreviations*: GTV, gross tumor volume; ITV, interval tumor volume; PTV, planning target volume

Using univariate analyses, the significant factors related to LFFS were tumor size (*p* = 0.006), GTV (*p* = 0.003), ITV (*p* < 0.001), PTV (*p* < 0.001), and PTV D2 (*p* = 0.030) (S2 Table). Using multivariate analyses adjusted for sex, age, primary cancer, tumor site, oligometastatic status, number of lesions treated per SBRT session, and tumor size or volume (Table 5), PTV D2 was the sole dosimetric parameter significantly associated with LFFS. Other dosimetric parameters, such as PTV D95, D98, and Dmean were not significantly related to LFFS. A larger tumor size and greater GTV, ITV, and PTV were significantly associated with worse LFFS.

**Table 5:** Multivariate analyses of risk factors for local failure-free survival after SBRT considering the BED10 of PTV D2.

		Multivariate Analysis		
		HR	(95% CI)	p
Primary cancer	Rectum vs. Colon	2.85	(0.91–8.95)	0.073
Tumor size (mm)	^3^ 11 mm vs. < 11 mm	5.43	(1.52–19.42)	0.009
GTV volume	^3^ 1.0 cc vs. > 1.0 cc			
ITV volume	^3^ 1.9 cc vs. < 1.9 cc			
PTV volume	^3^ 10.2 cc vs. < 10.2 cc			
PTV D2	^3^ 159 Gy_10_ vs. < 159 Gy_10_	0.31	(0.10–0.97)	0.044

**Fig 3:**
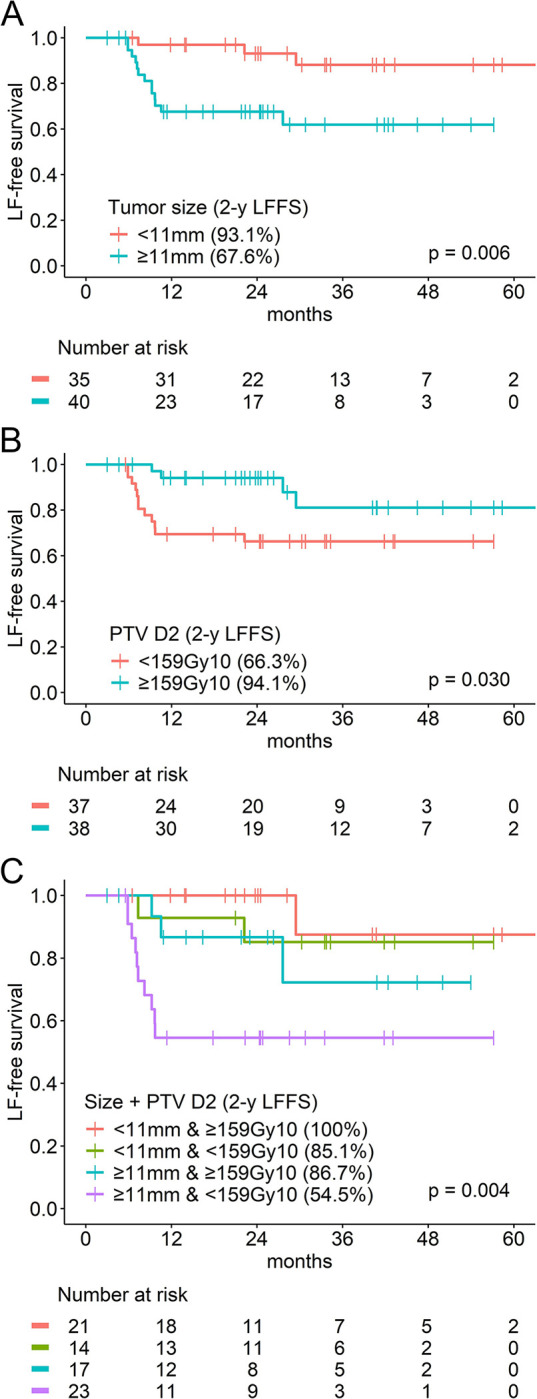
Local failure-free survival. Local failure-free survival curves of (A) tumor size, (B) BED10 of PTV D2, and (B) the combination of tumor size and BED10 of PTV D2.

Tumors equal to or larger than the median size (*p* = 0.006) and those receiving < 159 Gy_10_ of PTV D2 (*p* = 0.030) showed significantly lower LFFS (Fig 3A and 3B). Tumors equal to or larger than the median size/volume yet receiving < 159 Gy_10_ of PTV D2 showed the lowest LFFS (Fig 3C). Similar outcomes were observed in GTV, ITV, and PTV (S2A–S2F Fig). However, this trend was not observed in the combination of tumor size and PTV D95 (S2G Fig).

## Discussion

In this study using PTV dose parameters calculated using Monte Carlo-based calculation algorithms, tumor size, target volumes, and maximum dose represented by PTV D2 were significant factors for local control after SBRT in patients with lung and liver metastases from colorectal cancer. Tumors equal to or larger than the median size/volume and receiving < 159 Gy_10_ (median PTV D2) showed the lowest LFFS.

Higher prescription doses are recommended to improve the local control of metastatic colorectal cancer. This study included oligometastatic tumors treated with prescription doses of ≥100 Gy_10_, which is the dose level recommended in previous studies [9–12]. Although tumors receiving ≥ 120–132 Gy_10_ or <150 Gy_10_ had achieved a higher local control in some studies [13–17], BED10 of PTV D95 was not associated with local control in this study, where the median of BED10 of PTD D95 was 113 (106–120) Gy_10_. Moreover, PTV D98 did not correlate with local control. A secondary analysis of the SABR-COMET phase II trial showed that lesional control after SBRT was not influenced by a compromise of target coverage with an analysis using the coverage compromise index being defined as the minimum dose/prescription dose, where the minimum dose referred to the dose covering 99% of the PTV [21].

As observed in previous clinical studies, the closer correlation of Dmax and PTV D2 with local control, compared with other dose parameters, is likely due to the characteristics of SBRT planning [22, 23]. Considering lung SBRT, where dose fall-off is critical, the dose distribution has a steep gradient. This means that prescribed doses do not directly reflect the actual dose delivered to the primary tumor but instead represent the lowest dose within the PTV. A higher maximum dose has been shown to correlate more closely with local control. Similarly, in regard to liver SBRT, Thaper et al. demonstrated that in an inhomogeneous treatment plan, using calculations based on the Niemierko and Poisson models, increasing the maximum dose improves tumor control probability without significantly increasing the risk of complications in normal tissues [24].

PTV D2 of ≥159 Gy_10_ was significantly associated with better local control in this study. Similarly, BED10 at the isocenter (BED10isocenter) was related to local control in studies enrolling metastatic tumors from various cancers: the BED10isocenter to achieve 90% tumor control probability in patients with lung metastases was 160 Gy_10_ [25], and the BED10isocenter of 150 Gy_10_ was a discriminator for metastatic tumor control with a local control of > 80% in liver metastases cases [26]. Moreover, Klement et al. [27] estimated that the BED10isocenters to achieve ≥ 90% local control at 2 years were 187 Gy_10_ without prior chemotherapy and 300 Gy_10_ with prior chemotherapy for colorectal metastases. Based on these findings, prescribing ≥ 100 Gy_10_ to cover 95% of the PTV with maximum doses of > 150–160 Gy_10_ might be a good strategy while adjusting PTV coverage to meet organ-at-risk constraints. Although dose escalation is useful for enhancing local control, the minimum necessary dose should be used to preserve lung and liver functions due to the high frequency of new metastases in the lungs and liver, requiring repeated local therapies.

Differences in dose distributions among different dose calculation algorithms have been extensively studied for lung SBRT, mostly reporting that type-A (e.g., PBC) or type-B (e.g., AAA) algorithms tend to overestimate the PTV dose indices, particularly near the PTV margin [19, 28, 29]. When comparing PTV D98, D95, D50, and D2 between the AAA and MC dose distributions in the lung SBRT plans, the average differences were 6.5 ± 9.6%, 5.9 ± 7.2%, 2.1 ± 3.1%, and –0.6 ± 1.2%, respectively [28]. In the same study, the differences in PTV dose indices between AXB and MC dose distributions were less, with an average difference of 2.6 ± 4.6 to 0.6 ± 1.4%. Zhou et al. [19] revealed that PTV D95 was reduced by 2.3 ± 4.4% (up to 16.7%) when lung SBRT plans based on the AAA algorithm were recalculated using the AXB algorithm with identical plan parameters, along with a larger compromise in PTV coverage in smaller tumors. This complicates the determination of the optimal prescription dose. In a study revealing a difference of 9.2 ± 7.6% in PTV D95 between AAA- and MC-based calculations, dosimetric parameters calculated by the AAA were not associated with LF, with ITV D1 calculated by AXB being the sole significant factor for LF [29]. Nevertheless, most of the aforementioned studies did not specify the dose calculation algorithms and prescription policies used when investigating the relationship between prescription doses and local control. Given the significant variations in SBRT techniques and dose prescriptions across institutions, standardized SBRT planning and dose prescriptions that reflect actual tumor doses are crucial for providing optimal dose-fractionation guidelines [30, 31].

Our study found that a larger PTV of ≥ 10.2 cc showed a poorer local control compared with smaller tumors using multivariate analysis (hazard ratio, 10.92; 95% confidence interval, 2.39–49.82; *p* = 0.002). A greater tumor size of ≥ 11 mm led to worse 2-year LFFS rate (93.1% in < 11 mm and 67.6% in ≥ 11 mm, p = 0.006, Fig 3A). These findings are consistent with those of previous studies showing that a larger tumor volume was associated with inferior local control after SBRT for metastatic lesions. Qiu et al. [32] found that a GTV > 14.1 cc showed significantly lower LFFS, with most patients receiving 50 Gy in 10 fractions or 50 Gy in five fractions. Jung et al. [33] revealed that a GTV of > 1.5 cc was related to lower local control in patients receiving mostly 48 Gy or 60 Gy in four fractions. Although previous studies did not analyze the correlation between tumor size and radiation dose, larger tumors might have received lower doses owing to the dose constraints of the surrounding critical organs. In the current study, larger tumors received a lower PTV D2, without difference in PTV D95 (S3A Fig). Furthermore, even among larger tumors, those with PTV D2 < 159 Gy_10_ showed worse LFFS compared with those receiving at least 159 Gy_10_ to PTV D2. Considering the effects of the combination of tumor size/volume and PTV D2 on LFFS, with the lowest local control in larger tumors with PTV D2 < 159 Gy_10_ (Fig 3 and S2 Fig), increasing the maximum dose would be particularly beneficial for larger tumors rather than increasing PTV D95.

The relationship between metastatic location and local control in patients with metastatic colorectal cancers has been reported in previous studies, with liver metastases being an unfavorable factor for local control after SBRT compared with lung metastases [13, 27, 34]. Ahmed et al. [34] reported a significant difference in the 2-year LFFS after 60 Gy in five fractions of SBRT between lung and liver metastases with similar tumor sizes (100% vs. 73.0%, *p* = 0.026), despite the small cohort size of 29 total lesions. Similarly, a study of 388 patients with 500 metastatic lesions followed up for a median of 12.1 months showed that LF occurred more frequently in patient with liver than in those with lung metastases [27]. Although liver metastases tended to have a lower LFFS compared with lung metastases (55.6% vs. 83.3% at 2 years, *p* = 0.068) in this study, tumor site was not a significant factor using the multivariate analysis. This may be explained by the liver tumors being larger and receiving lower PTV D2 without differences in PTV D98, D95, and Dmean (S3 Fig and S3 Table). Our findings do not exclude the possibility of differences in radiosensitivity between liver and lung metastases. However, investigating whether prioritizing a higher maximum dose over the prescribed dose in liver SBRT planning could enhance local control would be valuable, as suggested by the overall findings of this study.

A history of chemotherapy before SBRT has been a negative factor for local control in some studies [11, 27, 35]. However, data on local control after SBRT according to the type of oligometastatic disease, which considers previous or current use of chemotherapy at the time of diagnosis, are scarce. In this study, the local control did not differ among de novo, repeat, or induced oligometastatic diseases. When dividing lesions into oligometastases (metastatic tumors identified at the time of diagnosis or recurrence) and oligoprogression (metastatic lesions that progressed during or at the end of the systemic therapy) as the study by Lee at al. [16], no difference was observed in the same analyses performed for oligometastatic type (Table 4). This consistency aligns with the findings reported in their study.

There are some limitations owing to the retrospective nature of this study and the small number of patients. First, not all LFs may have been captured because of the limited follow-up of patients with poor performance statuses or those experiencing disease progression at other sites. Second, the results of this study may not be applicable to larger tumors, as three-quarters of the lesions measured ≤ 17 mm in size. Third, SBRT details were heterogeneous in terms of planning and delivery techniques, prescription methods, and dose-fractionation schedules. Thus, the current study used PTV doses calculated with Monte Carlo-based algorithms, which provided estimates closer to the actual tumor doses in all analyses.

In conclusion, among various dosimetric parameters, PTV D2 was identified as the most important factor affecting local control after SBRT for lung or liver oligometastatic disease from colorectal cancer. Escalating the maximum dose may be necessary during SBRT planning, particularly for larger tumors. Finally, prospective studies with large cohorts are imperative to determine optimal SBRT planning and prescription guidelines for oligometastatic colorectal cancer.

## Supporting information

S1 Table Dose-fractionation schedules according to the tumor location. (PDF)

S2 Table Univariable analyses of risk factors for local failure-free survival after SBRT. (PDF)

S3 Table Differences in characteristics of lung and liver metastases. (PDF)

S1 Fig Correlation among PTV doses. Red dots indicate lesions showing local failure. Tumor locations are shown as circles for the lung and triangles for the liver.
(PDF)

S2 Fig Local failure-free survival curves. Local failure-free survival curves of (A) GTV volume, (B) GTV volume and PTV D2, (C) ITV volume, (D) ITV volume and PTV D2, (E) PTV volume, (F) PTV volume and PTV D2, and (G) tumor size and PTV D95.
(PDF)

S3 Fig Target volumes and the dose distribution. Target volumes and the dose distribution of PTV. (A, B) Tumor size, (C, D) GTV volume and (E, F) PTV volume. Dotted lines represent the values of quartiles values of each variable. Red dots indicate lesions showing local failure. Tumor locations are shown as circles for the lung and triangles for the liver.
(PDF)

S1 Dataset (XLSX)
