# Supplementary material for: Effects of maximum dose on local control after stereotactic body radiotherapy for oligometastatic tumors of colorectal cancer
Source: PLoS One. 2025 Jan 3;20(1):e0313438. doi: 10.1371/journal.pone.0313438 (PMC11698420; doi:10.1371/journal.pone.0313438)
Supplement: S1 Table — (PDF) [file pone.0313438.s001.pdf]

S1 Table. Dose-fractionation schedules according to the tumor location.

Daily dose	Fractions	Lung (N = 65)	Liver (N = 10)	BED10 (Gy_{10})
20.0	3	1	0	180.0
18.0	3	5	0	151.2
15.0	4	2	0	150.0
17.0	3	2	0	137.7
16.0	3	1	5	124.8
13.0	4	15	1	119.6
12.5	4	17	0	112.5
15.0	3	0	1	112.5
12.0	4	14	1	105.6
7.5	8	1	0	105.0
10.0	5	7	2	100.0
