# Supplementary material for: Effects of maximum dose on local control after stereotactic body radiotherapy for oligometastatic tumors of colorectal cancer
Source: PLoS One. 2025 Jan 3;20(1):e0313438. doi: 10.1371/journal.pone.0313438 (PMC11698420; doi:10.1371/journal.pone.0313438)
Supplement: S2 Table — (PDF) [file pone.0313438.s002.pdf]

**S2 Table.** Univariable analyses of risk factors for local failure-free survival after SBRT.

| | Patients | 2-y LFFS | p |
|---|---|---|---|
| Age | | | 0.766 |
| ≤ 70 years | 38 (50.7%) | 83.0% | |
| > 70 years | 37 (49.3%) | 80.0% | |
| Sex | | | 0.386 |
| Male | 49 (65.3%) | 81.3% | |
| Female | 26 (34.7%) | 76.2% | |
| Primary cancer | | | 0.199 |
| Colon | 31 (41.3%) | 85.4% | |
| Rectum | 44 (58.7%) | 79.1% | |
| Tumor site | | | 0.068 |
| Lung | 65 (86.7%) | 83.3% | |
| Liver | 10 (13.3%) | 55.6% | |
| Oligometastatic disease* | | | 0.837 |
| De novo | 27 (36.0%) | 80.8% | |
| Repeat | 37 (49.3%) | 79.7% | |
| Induced | 11 (14.7%) | 75.0% | |
| Multiplicity at the time of treatment | | | 0.744 |
| Single | 37 (49.3%) | 79.4% | |
| Multiple | 38 (50.7%) | 80.1% | |
| Tumor size (mm) | | | 0.006 |
| < 11 mm | 35 (46.7%) | 93.1% | |
| ≥ 11 mm | 40 (53.3%) | 67.6% | |
| GTV volume | | | 0.003 |
| > 1.0 cc | 37 (49.3%) | 90.7% | |
| ≥ 1.0 cc | 38 (50.7%) | 68.6% | |
| ITV volume | | | <0.001 |
| < 1.9 cc | 37 (49.3%) | 97.1% | |
| ≥ 1.9 cc | 38 (50.7%) | 61.8% | |
| PTV volume | | | <0.001 |

| | | | |
|---|---|---|---|
| < 10.2 cc | 37 (49.3%) | 97.1% | |
| ≥ 10.2 cc | 38 (50.7%) | 62.5% | |
| PTV D2 | | | 0.030 |
| < 159 $Gy_{10}$ | 37 (49.3%) | 66.3% | |
| ≥ 159 $Gy_{10}$ | 38 (50.7%) | 94.1% | |
| PTV D95 | | | 0.562 |
| < 113 $Gy_{10}$ | 37 (49.3%) | 73.8% | |
| ≥ 113 $Gy_{10}$ | 38 (50.7%) | 85.7% | |
| PTV D98 | | | 0.294 |
| < 106 $Gy_{10}$ | 37 (49.3%) | 73.8% | |
| ≥ 106 $Gy_{10}$ | 38 (50.7%) | 85.7% | |
| PTV Dmean | | | 0.124 |
| < 135 $Gy_{10}$ | 37 (49.3%) | 69.2% | |
| ≥ 135 $Gy_{10}$ | 38 (50.7%) | 91.2% | |

LFFS, local failure-free survival; GTV, gross tumor volume; ITV, interval tumor volume; PTV, planning target volume

*Oligometastatic disease was classified according to European Society for Radiotherapy and Oncology (ESTRO)/European Organization for the Research and Treatment of Cancer (EORTC) classification (Guckenberger M, et al. Lancet Oncol. 2020;21:e18-e28.).
