# Supplementary material for: Effects of maximum dose on local control after stereotactic body radiotherapy for oligometastatic tumors of colorectal cancer
Source: PLoS One. 2025 Jan 3;20(1):e0313438. doi: 10.1371/journal.pone.0313438 (PMC11698420; doi:10.1371/journal.pone.0313438)
Supplement: S3 Table — (PDF) [file pone.0313438.s003.pdf]

S3 Table. Differences in characteristics of lung and liver metastases.

	Liver (N = 10)	Lung (N = 65)	p
Tumor size (mm)	16 [15;20]	10 [6;16]	0.003
GTV volume (cc)	6.5 [4.0;12.9]	0.7 [0.3;2.2]	<0.001
ITV volume (cc)	9.3 [5.7;15.7]	1.1 [0.7;4.1]	<0.001
PTV volume (cc)	29.2 [18.9;38.0]	9.6 [6.2;17.6]	0.001
PTV D2 (Gy_{10})	137 [116;151]	163 [151;172]	<0.001
PTV D95 (Gy_{10})	114 [97;119]	113 [106;120]	0.635
PTV D98 (Gy_{10})	109 [93;115]	106 [100;113]	0.797
PTV Dmean (Gy_{10})	131 [114;140]	136 [129;144]	0.097

GTV, gross tumor volume; ITV, interval tumor volume; PTV, planning target volume
