# Supplementary material for: Effects of maximum dose on local control after stereotactic body radiotherapy for oligometastatic tumors of colorectal cancer
Source: PLoS One. 2025 Jan 3;20(1):e0313438. doi: 10.1371/journal.pone.0313438 (PMC11698420; doi:10.1371/journal.pone.0313438)

S1 Fig. Correlation among PTV doses. Red dots indicate lesions showing local failure. Tumor locations are shown as circles for the lung and triangles for the liver.

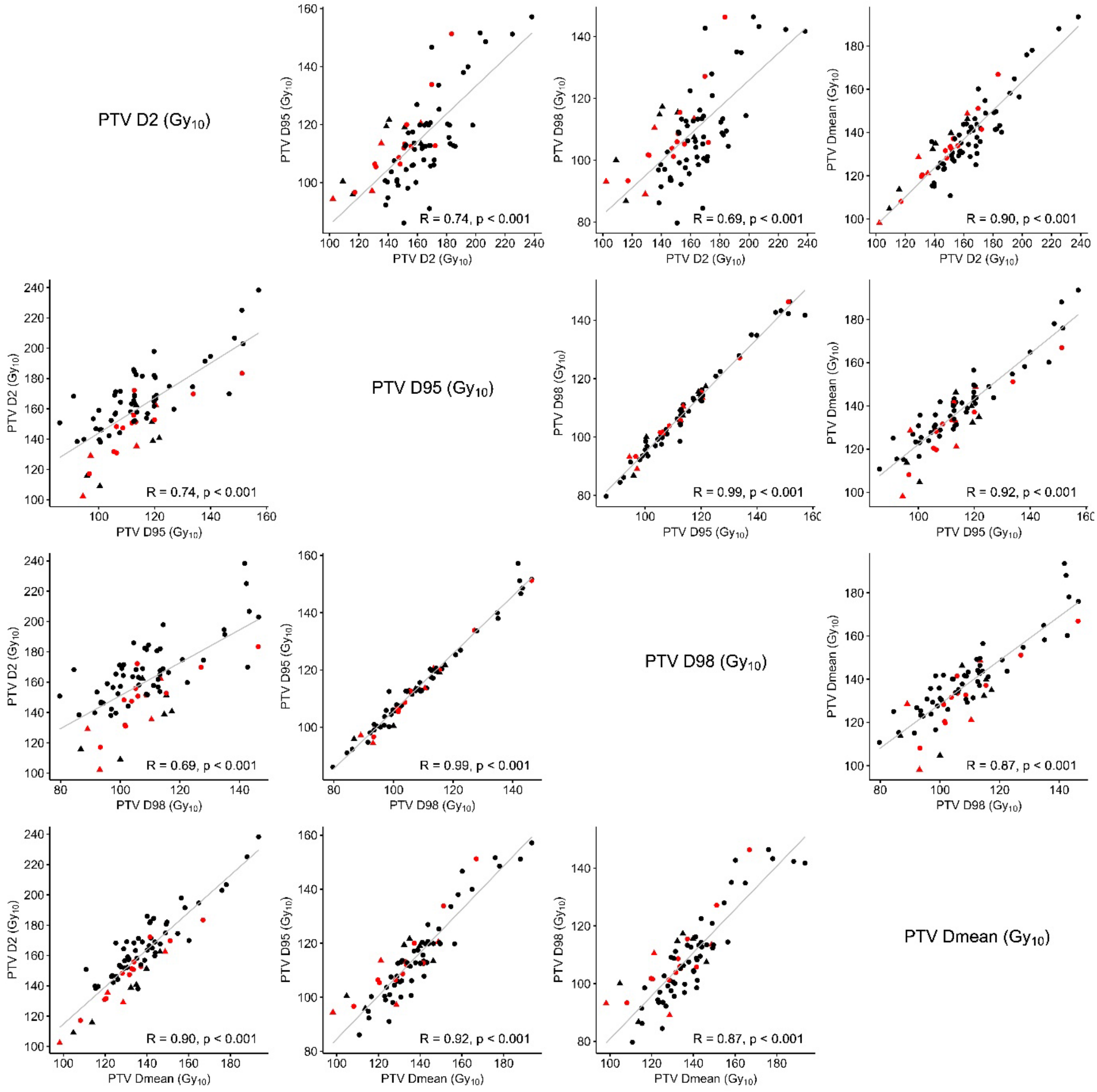

Supplement: S1 Fig — Red dots indicate lesions showing local failure. Tumor locations are shown as circles for the lung and triangles for the liver. (PDF) [file pone.0313438.s004.pdf]
