# Supplementary material for: Effects of maximum dose on local control after stereotactic body radiotherapy for oligometastatic tumors of colorectal cancer
Source: PLoS One. 2025 Jan 3;20(1):e0313438. doi: 10.1371/journal.pone.0313438 (PMC11698420; doi:10.1371/journal.pone.0313438)
Supplement: S2 Fig — Local failure-free survival curves of (A) GTV volume, (B) GTV volume and PTV D2, (C) ITV volume, (D) ITV volume and PTV D2, (E) PTV volume, (F) PTV volume and PTV D2, and (G) tumor size and PTV D95. (PDF) [file pone.0313438.s005.pdf]

**S2 Fig. Local failure-free survival curves.** Local failure-free survival curves of (A) GTV volume, (B) GTV volume and PTV D2, (C) ITV volume, (D) ITV volume and PTV D2, (E) PTV volume, (F) PTV volume and PTV D2, and (G) tumor size and PTV D95.

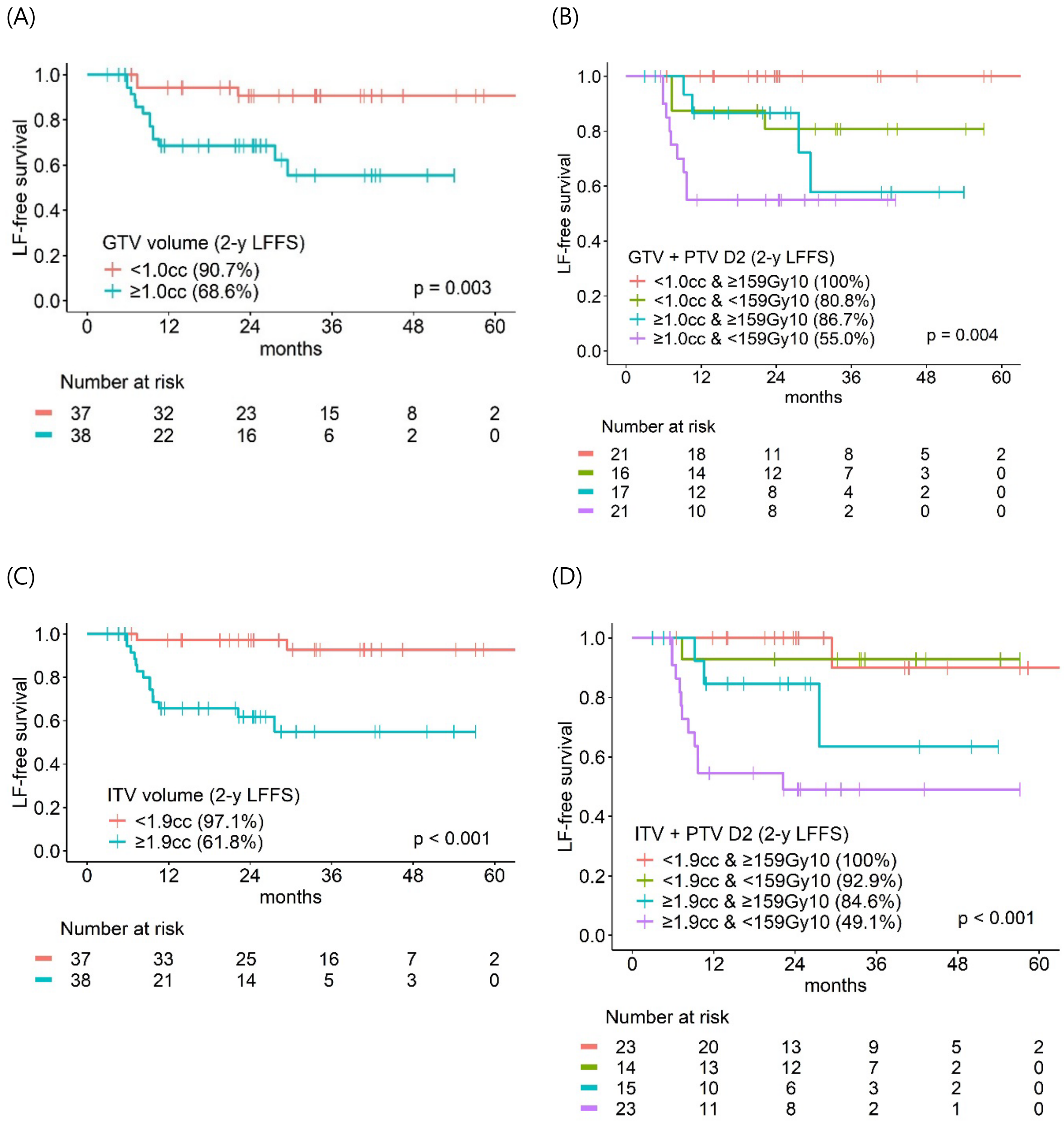

(E)

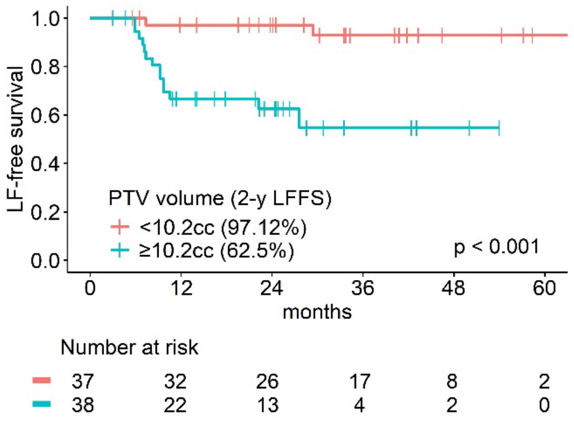

1.0
0.8
0.6
0.4
0.2
0.0
LF-free survival
PTV volume (2-y LFFS)
<10.2cc (97.12%)
≥10.2cc (62.5%)
p < 0.001
0
12
24
36
48
60
months
Number at risk
37 32 26 17 8 2
38 22 13 4 2 0


(F)

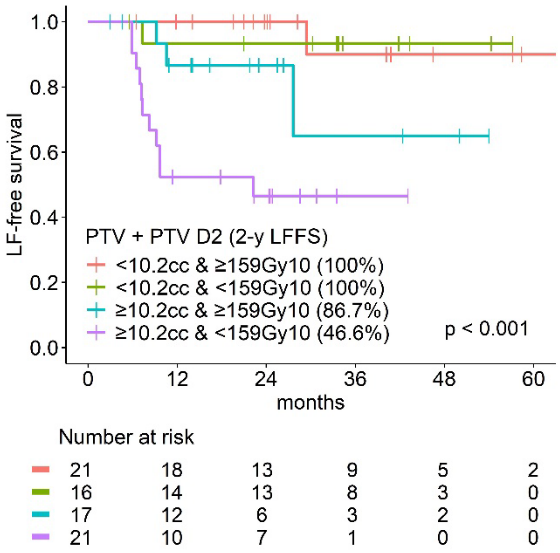

1.0
0.8
0.6
0.4
0.2
0.0
LF-free survival
PTV + PTV D2 (2-y LFFS)
<10.2cc & ≥159Gy10 (100%)
<10.2cc & <159Gy10 (100%)
≥10.2cc & ≥159Gy10 (86.7%)
≥10.2cc & <159Gy10 (46.6%)
p < 0.001
0
12
24
36
48
60
months
Number at risk
21 18 13 9 5 2
16 14 13 8 3 0
17 12 6 3 2 0
21 10 7 1 0 0


(G)

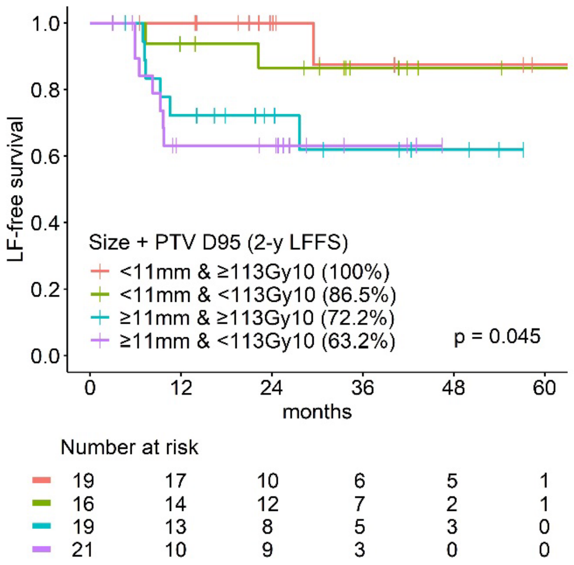

1.0
0.8
0.6
0.4
0.2
0.0
LF-free survival
Size + PTV D95 (2-y LFFS)
<11mm & ≥113Gy10 (100%)
<11mm & <113Gy10 (86.5%)
≥11mm & ≥113Gy10 (72.2%)
≥11mm & <113Gy10 (63.2%)
p = 0.045
0
12
24
36
48
60
months
Number at risk
19 17 10 6 5 1
16 14 12 7 2 1
19 13 8 5 3 0
21 10 9 3 0 0
