# Supplementary material for: Effects of maximum dose on local control after stereotactic body radiotherapy for oligometastatic tumors of colorectal cancer
Source: PLoS One. 2025 Jan 3;20(1):e0313438. doi: 10.1371/journal.pone.0313438 (PMC11698420; doi:10.1371/journal.pone.0313438)

S3 Fig. Target volumes and the dose distribution. Target volumes and the dose distribution of PTV. (A, B) Tumor size, (C, D) GTV volume and (E, F) PTV volume. Dotted lines represent the values of quartiles values of each variable. Red dots indicate lesions showing local failure. Tumor locations are shown as circles for the lung and triangles for the liver.

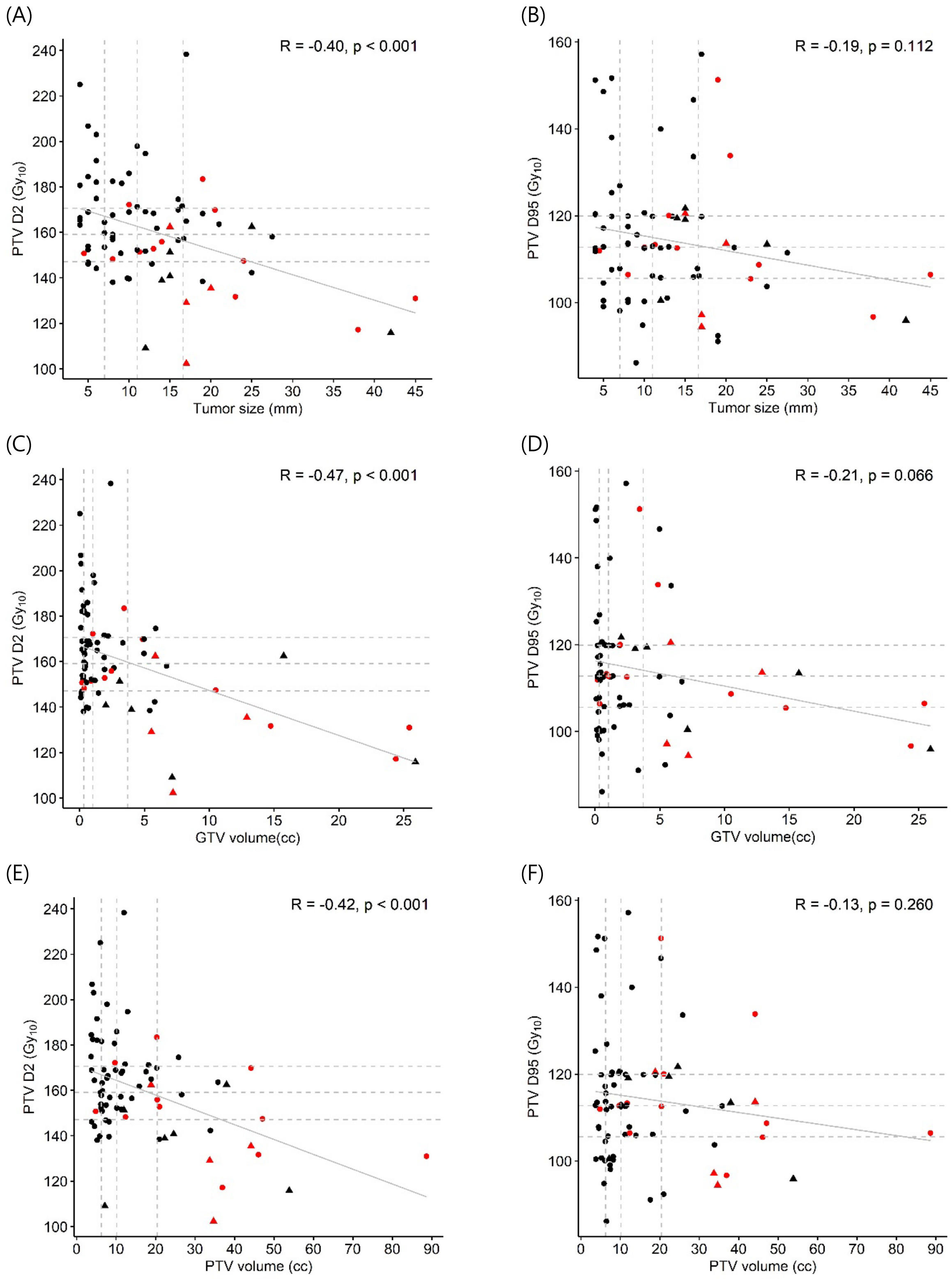

Supplement: S3 Fig — Target volumes and the dose distribution of PTV. (A, B) Tumor size, (C, D) GTV volume and (E, F) PTV volume. Dotted lines represent the values of quartiles values of each variable. Red dots indicate lesions showing local failure. Tumor locations are shown as circles for the lung and triangles for the liver. (PDF) [file pone.0313438.s006.pdf]
